# Pancreatic Cysts

**Published:** 1904-07-02

**Authors:** 


### PANCREATIC CYSTS.

It is only of recent years that abnormal conditions of the pancreas can be said to have been at all generally recognised as clinical events capable of diagnosis and treatment. The work of Mayo Robson in this direction has opened up a wide field and has demonstrated the necessity of recognising various forms of pancreatic disease among the possible causes of abdominal symptoms. The diagnosis of disease of the pancreas even yet, it must be admitted, is not in a very confident position and in many instances has only been made as the result of an exploratory operation. When, apart from other diseased conditions, attention is paid solely to cysts of the pancreas, it must be again admitted that it is not easy to present any very clear-cut rules for the clinical recognition of these. Thus they may vary in size within extremely wide limits, and though at one time considerable stress was laid upon the presence of dark or blood-stained fluid as a point in the diagnosis, it is now known that the fluid may have characters quite the opposite of these. Indeed, a case was reported some four years ago by Dr. Sidney Phillips in which a cyst of pancreatic origin not only grew to such an enormous size as to simulate ascites, but contained a clear fluid of a pale yellow colour with a specific gravity of 1002, and free from all evidences of blood coloration and deposit.

In short, reviewing the whole position, it must be admitted that among the many perplexities which the practitioner has to encounter in the region of abdominal diagnosis, cysts of the pancreas are deserving of special mention. Thus it is all the more necessary to utilise all information which can have a bearing on the practical problem, and an observation recorded by Mr. R. H. Parry, of the Victoria Hospital, Glasgow, seems to be of distinct value in this direction. According to Mr. Parry's experience, cysts of the pancreas are usually found in the "tail" of the organ, and therefore, when large enough to demand clinical investigation, the cyst presents itself as a tumour in the left half of the upper abdomen. In such a position it is apt to be mistaken for—and, as a matter of fact, often is mistaken for—a movable or floating kidney. It is, of course, easy to say that a pancreatic cyst never has such a degree of mobility as may exist in the case of a movable kidney, but the fact remains that it is slightly movable both in the vertical and lateral direction, and that an estimation of the extent to which an abdominal tumour is movable is much more difficult in practice than theoretical considerations taken alone would suggest. Hence it is not altogether surprising to find that every now and then a tumour in the left abdomen is called a floating kidney, only sooner or later to be discovered as a cyst of the pancreas. There are two facts of observation which should be of assistance in preventing this mistake. In the first place, it will be remembered that mobility of the kidney, though not very uncommon on the right side, is decidedly exceptional on the left side, and still more exceptional is it to find that the left kidney alone has this abnormal quality. In dealing with the problems of clinical medicine unqualified propositions are rarely sustained by experience, yet it may be safely said that mobility of the left kidney with a fixed and normal right kidney is an extremely rare, and, some would even say, an unprecedented, event. Therefore, when a tumour having more or less mobility is found in the left abdomen and the right kidney shows no abnormal excursion in response to the usual tests, a suggestion to name such a tumour a movable left kidney should be received with considerable hesitation. The fact that there is not a movable right kidney is sufficient in itself to render the diagnosis suspect. Again, in mobility of the right kidney, it will be remembered that re-position of the viscus generally leaves a small portion of the lower part accessible to palpation by the bi-manual method. But in the case of the left kidney this is not so. The normal anatomical position of the left kidney is at a somewhat higher level than that of the right, and even when it has to some extent loosed its moorings, the organ, when restored to its proper place, ceases any longer to be within reach of the palpating hand. Hence a movable tumour in the left abdomen which cannot be manipulated towards the normal position of the left kidney in such a fashion as to cause its practical disappearance from the field of examination is almost certainly not the left kidney. A cyst of the pancreas, at least one arising from the "tail" of that organ, exactly fulfils this description; it always refuses completely to disappear under manipulation, but, on the contrary, leaves in the region of the loin a definite sense of tumour which cannot be forced upwards under the vault of the diaphragm. These two considerations, therefore, (1) the non-mobility of the left kidney unless the right is also similarly affected, and (2) the effect of manipulation upon the persistence or non-persistence of a palpable tumour are of value in the diagnosis of pancreatic cyst to the extent that they help to prevent this condition being mistaken for movable left kidney. Of course there are other possibilities, such, for example, as an enlarged spleen or a tumour growing in the wall of the stomach, that have to be considered. In each of these there may be mobility, and in each also no manipulation will cause the tumour completely to disappear.

The facts now suggested are not offered as a conclusive basis for a positive diagnosis of pancreatic cyst. But they do accomplish two ends. First they end in the confident exclusion of movable kidney—a diagnosis which has on more than one occasion been applied to cyst of the pancreas. And secondly, they impress upon the mind the necessity of considering this last-mentioned condition when a tumour with more or less mobility is found in the upper abdomen and to the left of the middle line.

